# Epitope Mapping of M36, a Human Antibody Domain with Potent and Broad HIV-1 Inhibitory Activity

**DOI:** 10.1371/journal.pone.0066638

**Published:** 2013-06-11

**Authors:** Chao Wan, Jianping Sun, Weizao Chen, Xiaohui Yuan, Huihui Chong, Ponraj Prabakaran, Dimiter S. Dimitrov, Yuxian He

**Affiliations:** 1 MOH Key Laboratory of Systems Biology of Pathogens and AIDS Research Center, Institute of Pathogen Biology, Chinese Academy of Medical Sciences and Peking Union Medical College, Beijing, China; 2 Protein Interactions Group, CCRNP, CCR, National Cancer Institute, National Institutes of Health, Frederick, Maryland, United States of America; Shanghai Medical College, Fudan University, China

## Abstract

M36 is the first member of a novel class of potent HIV-1 entry inhibitors based on human engineered antibody domains (eAds). It exhibits broad inhibitory activity suggesting that its CD4-induced epitope is highly conserved. Here, we describe fine mapping of its epitope by using several approaches. First, a panel of mimotopes was affinity-selected from a random peptide library and potential m36-binding residues were computationally predicted. Second, homology modeling of m36 and molecular docking of m36 onto gp120 revealed potentially important residues in gp120-m36 interactions. Third, the predicted contact residues were verified by site-directed mutagenesis. Taken together, m36 epitope comprising three discontinuous sites including six key gp120 residues (Site C1: Thr123 and Pro124; Site C3: Glu370 and Ile371; Site C4: Met426 and Trp427) were identified. In the 3D structure of gp120, the sites C1 and C4 are located in the bridging sheet and the site C3 is within the β15-α3 excursion, which play essential roles for the receptor- and coreceptor-binding and are major targets of neutralizing antibodies. Based on these results we propose a precise localization of the m36 epitope and suggest a mechanism of its broad inhibitory activity which could help in the development of novel HIV-1 therapeutics based on eAds.

## Introduction

The epidemic of HIV-1 infection continues to be an unabated worldwide problem in the absence of an effective vaccine. Highly active antiretroviral therapy (HAART) using mainly reverse-transcriptase and protease inhibitors has dramatically decreased morbidity and mortality among people living with HIV-1. Several HIV-1 entry inhibitors including the fusion inhibitor T20 (Enfuvirtide, Fuzeon) and the coreceptor CCR5 antagonist Maraviroc (Selzentry) have also been approved by the US FDA and are used especially in those patients who fail to respond to HAART [1]. However, the success of treatment is frequently limited by serious adverse effects and the emergence of drug-resistant HIV-1 mutants[2]. Thus, there is an urgent need to develop new classes of inhibitors with different mechanisms of action, which when combined with the existing inhibitors, could exhibit increased antiviral potency, breadth, and durability to viral resistance.

As a class of natural inhibitors of HIV-1 entry, neutralizing monoclonal antibodies (nAbs) are potent and generally more specific (safer) than small molecule drugs, and have thus been extensively explored as candidate therapeutics and prophylactics[3]–[4]. However, HIV-1 has evolved a variety of strategies to escape neutralization by antibodies generated by the human immune system, such as the extreme variability of its envelope glycoproteins (Envs) and the steric occlusion of conserved neutralizing epitopes[5]–[6]. Indeed, several human broadly nAbs including b12, 2G12, 2F5, and 4E10 are highly effective against HIV-1 infection *in vitro* and can confer sterilizing protection in animal models, but their administration to HIV-1-infected humans has resulted in only modest antiviral effects[7]–[9]. The disappointing results are in contrast to the clinical benefits provided by the currently approved therapeutic antibodies for other diseases. Since 2009, new human broadly nAbs against HIV-1 have been identified by using novel selection approaches such as high-throughput B cell sorting and functional screening. These antibodies include VRC01 and VRC02 [10], which target the CD4-binding site (CD4bs), PG9 and PG16 [11], which are directed against the conserved regions of variable loops of gp120 preferentially expressed on trimeric Envs, the series of PGT antibodies [12], which bind to various novel epitopes on gp120, and 10E8, which is specific for the membrane-proximal external region (MPER) of gp41. They are on average more potent and broadly neutralizing than b12, 2G12, 2F5, and 4E10 *in vitro*, and therefore could be more promising for HIV-1 prevention or therapy. To date, however, there are not much data from *in vivo* experiments that could prove this possibility.

Antibody fragments of small size could be more effective than naturally occurring full-length antibodies because they could more easily gain access to the highly guarded conserved structures of HIV-1 Envs [13]–[16]. In line with this possibility is the finding that the Fab and scFv formats of CD4-induced (CD4i) antibodies such as X5 and 17b, which target the coreceptor-binding site of gp120, are generally superior to their IgG formats in neutralizing HIV-1[5]. We therefore hypothesized that further decreasing the sizes of antibody fragments to the smallest independently folded single antibody domains but maintaining high binding affinity could lead to exceptionally potent and broadly cross-reactive HIV-1 neutralizers. By panning a large, highly diversified library of human VH domain sequentially against two Envs from different HIV-1 isolates, we identified the first reported human VH against HIV-1, m36, which showed potent inhibitory activity against genetically diverse HIV-1 isolates. M36 was also active against about 90% of the viruses resistant to ibalizumab, a clinically tested broadly neutralizing mAb (bnmAb) directed against mainly the second extracellular domain of CD4 (http://www.retroconference.org/2012b/PDFs/436.pdf). In a humanized NOD/SCID/γcnull mice model, m36.4, an affinity-matured version of m36, provided sterilizing protection of four of six animals against intrasplenical challenge with high-titer HIV-1 (>1000 TCID50s) while extensive infection was detected in all four control animals (http://www.retroconference.org/2011/Abstracts/41951.htm). Interestingly, m36 was able to enhance binding of CD4bs broadly nAbs to recombinant HIV-1 gp120s, suggesting that m36-gp120 fusion proteins could be a novel type of candidate HIV-1 vaccine immunogens possibly better than gp120s alone[17].

In this study, we have finely characterized the m36 epitope by combining three powerful approaches, including the mimotope-based computational prediction, molecular docking, and site mutagenesis. Our results provide important information for understanding the mechanism of m36-mediated inhibition and for exploring the structures and functions of HIV-1 Envs. They will also help in the further development of m36 as a novel class of potentially promising HIV-1 entry inhibitors and components of vaccine immunogens.

## Results

### Selection of peptide mimotopes for m36

**Figure 1:**
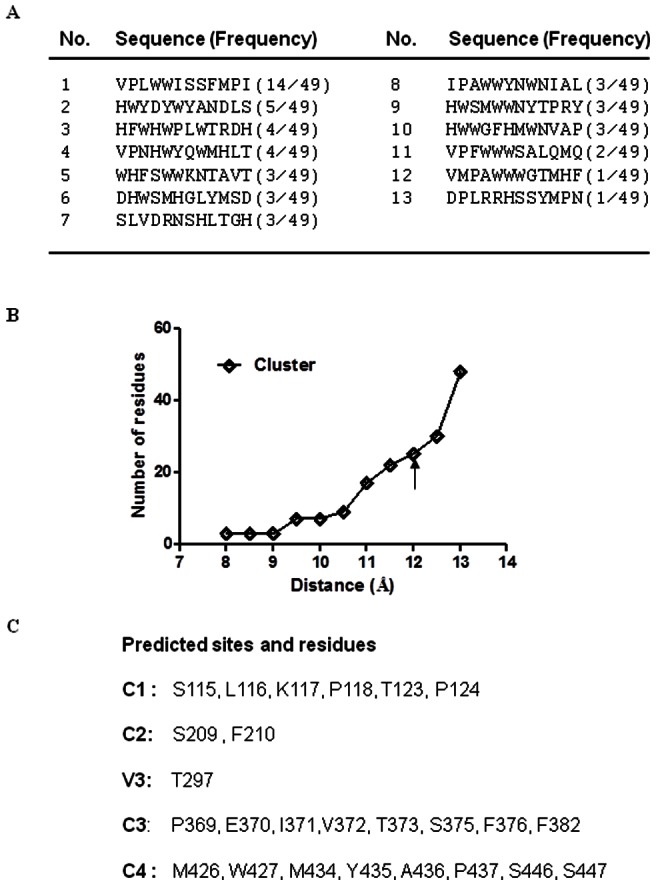
Computational prediction of m36-binding residues by mimotopes. **A.** Sequence of affinity-selected mimotopes from the peptide library. The selection frequency of a specific mimotope is shown in parentheses. **B.** The cluster Q-point identified by different values for the maximum distance. **C.** Predicted amino acid residues by Mapitope. The region and amino acid numbering are relative to HIV-1_HXB2_ gp120.

Our previous studies revealed that m36 targets a highly conserved CD4-induced structure that may overlap with the coreceptor-binding site of gp120 [18]–[19]. However, its binding motifs and residues have not been characterized. A number of studies demonstrated that mimotope-based prediction can serve as an ideal approach to map the conformational epitope of a given antibody[20]–[22]. Here, we sought to select the mimotopes for m36 from a 12-mer random peptide phage display library. After three rounds of biopanning, a total of 49 positive phage clones were isolated. The DNA sequencing of their inserts identified 13 different peptide mimotopes (Fig. 1A). Notably, one of the peptides (VPLWWISSFMPI) was predominately selected (14/49). However, we could not find apparent consensus sequences or significantly homologous motifs that can be used to deduce the binding sites or residues of m36, suggesting the confirmation-dependent nature of m36 epitope that is mostly exposed/formed after gp120 binding to CD4.

### Prediction of m36 epitope by Mapitope

Based on the selected mimotopes, we predicted the potential binding residues of m36 by the computational algorithm Mapitope, which is a powerful tool for mapping antibody epitopes[20]–[22]. The program ranks clusters and identifies better candidate epitopes by comparing the point of “quantum jump” which is defined as the “Q point.” A significant increase in the number of amino acids beyond the Q point may be resulted from the merging of adjacent clusters or recruitment of peripheral or underlying irrelevant amino acids, which leads to a sharp increase in the number of amino acids associated with a given D value. The selected 12-mer peptides were analyzed against a range of D values (8 to 13) and only one cluster was predicted. Since the D value would show a sharp rise above the residue number of the clusters and the cluster may have an adequate amount of residues, the Q point of 12-mer peptide library should be conducted at the point ST 12 (Fig. 1B). A total of 25 gp120 residues for the cluster were predicted and they were primarily located at the conserved regions of gp120 (Fig. 1C), including C1 (Ser115, Leu116, Lys117, Pro118, Thr123, Pro124), C2 (Ser209, Phe210), C3 (Pro369, Glu370, Ile371,Val372, Thr373, Ser375, Phe376, Phe382), and C4 (Met426, Trp427, Met434, Tyr435, Ala436, Pro437, Ser446, Ser447). As one exception, Thr297 was localized within the V3 loop. Apparently, several amino acid stretches overlap with or neighbor the receptor- or coreceptor-binding sites.

### Homology modeling of m36

**Figure 2:**
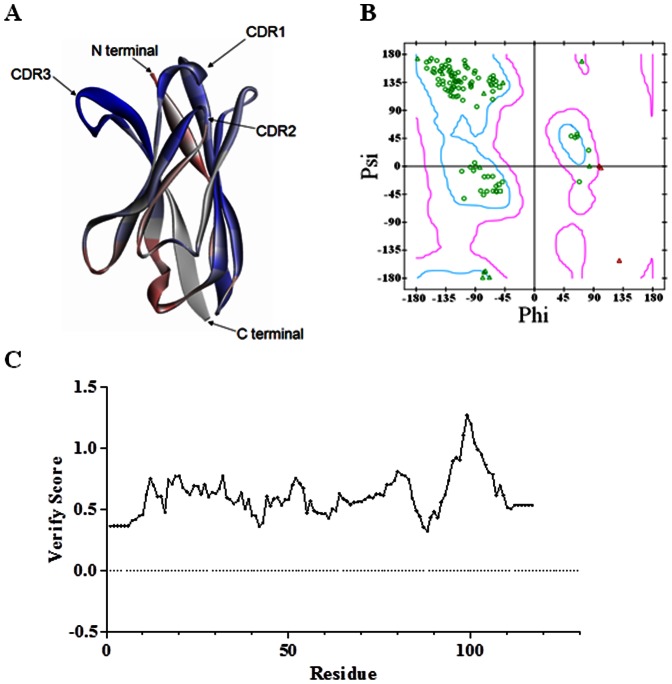
A Carton representation of the m36 model. **A.** Profile analysis of theoretical 3D model of m36. The color from blue to red indicates the high quality to the low quality of the model region. **B.** A Verify score of m36 model where all the values lie within the normal range. **C.** Ramachandran plot of the theoretical 3D model of m36.

It is conceivable that with a small size VH-based m36 might have better access to gp120 resides compared to antibodies with paired VH and VL[5]. To localize the key residues or binding sites targeted by m36, we chose to perform molecular docking study of gp120-m36 interactions. BLAST search analysis revealed that m36 has amino acid sequence similarity with three PDB entries (2GCY, 3E0T and 2NZ9) with 2GCY having the highest similarity although diminishing similarities for the CDRs. Based on the 2GCY crystal structure, an m36 model was developed with the help of the modeling program suite Discovery Studio and further refined using the ModLoop programs (Fig. 2A). The quality of the m36 structure was then assessed by RAMACHANDRAN plot (Fig. 2B). 87.5% of m36 amino acid residues are found in the allowed regions. Profile 3D verify score for the final VH model was 64.36, higher than the Verify expected high score (52.84), confirming the reliability of the modeled structure (Fig. 2C).

### M36 -binding interface on gp120 revealed by molecular docking

**Figure 3:**
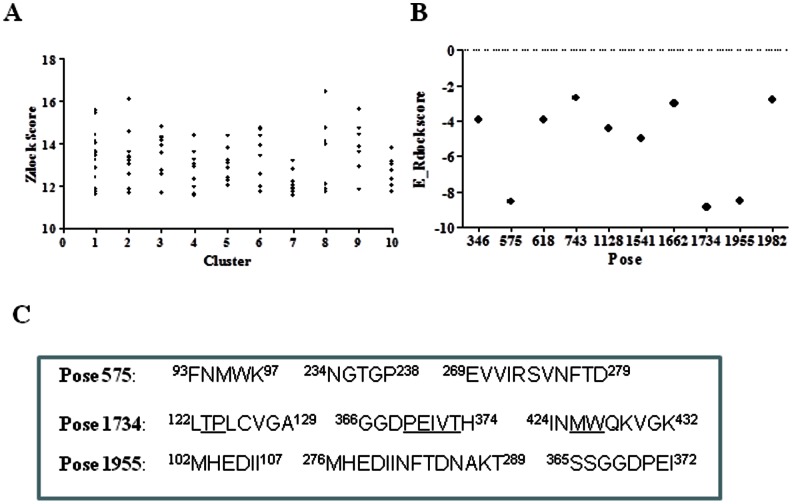
Molecular docking models of m36. **A**. Zdock score from gp120-m36 interaction simulations. **B.** E_Rock for the best poses from the result of ZDock. **C.** The interface sequence of the three top-score poses (575, 1734, 1955). The residues predicted by both Mapitope and molecular docking methods are underlined.

**Figure 4:**
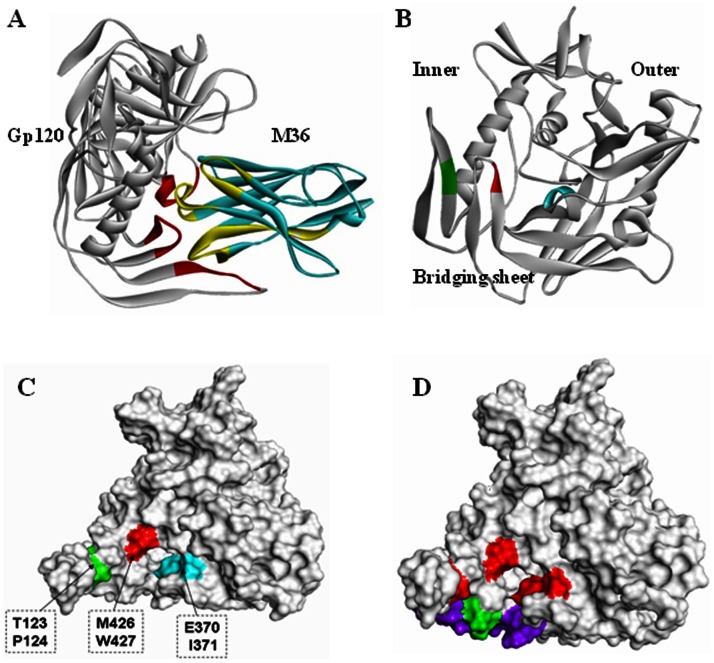
Localization of m36 epitope in gp120. **A.** Docking model for the pose 1734. The interface between gp120 and CDR of m36 are separately marked in red and yellow). **B.** Ribbon diagram of m36 binding sites on the 3D gp120 core structure. C1 site (Thr123, Pro124), C3 site (Glu370, Ile371) and C4 site (Met426, Trp427) are respectively colored in green, cyan and. **C.** Surface representation of m36 binding sites on gp120. **D.** Comparison of m36, 17b and X5 epitopes. M36 targets the CD4-binding face of the bridging sheet (red) while 17b (purple) and X5 (green) bind to the opposite face. The diagram was generated with Accelrys Discovery studio 3.5.

To gain insights into the interaction of m36 with gp120, we docked the m36 model with a gp120 crystal structure (2NXY) using the ZDOCK program. The top 100 complexes obtained were grouped into clusters according to their interactions. 88 models of the m36-gp120 complex with Zdock Scores above 11 were refined and re-ranked with the RDOCK program (Fig. 3A). The final RDOCK energy analysis indicated that pose 1734, which belongs to Cluster 1, has the lowest E_Rdock score (–8.84) (Fig. 3B). The E_Rdock scores of poses 575 (–8.54) and 1955 (–8.49) were similar to that of pose 1734. As shown in Fig. 3C, the three poses each contains three amino acid segments corresponding to drastically different domains of gp120, suggesting that Zdock may provide alternative and confounded outcomes. We observed that the amino acid stretches or motifs in pose 1734 are related to receptor or coreceptor binding and share the most common residues with those predicted by Mapitope. In contrast, the residues in the poses 575 and 1955 are not included in the binding sites of receptors or CD4i antibodies, implying that they might not participate in the interaction with m36. Therefore, we selected the pose 1734 for docking with m36 and further characterization. In the 3D structure of gp120 (Fig. 4A), the first segment of pose 1734 (aa122-129) overlaps with the β2 region in the V1V2 stem and the third segment (aa424-432) is located within the β20-β21 hairpin region. Both segments are involved in the formation of the bridging sheet between the outer and inner gp120 domains, which are critical for the binding of a coreceptor or CD4i antibodies. Particularly, the second segment (aa366-371) is within the β15-α3 excursion, which is a portion of the CD4 binding loop.

### Identification of gp120 residues critical for m36 binding by point-mutagenesis

**Figure 5:**
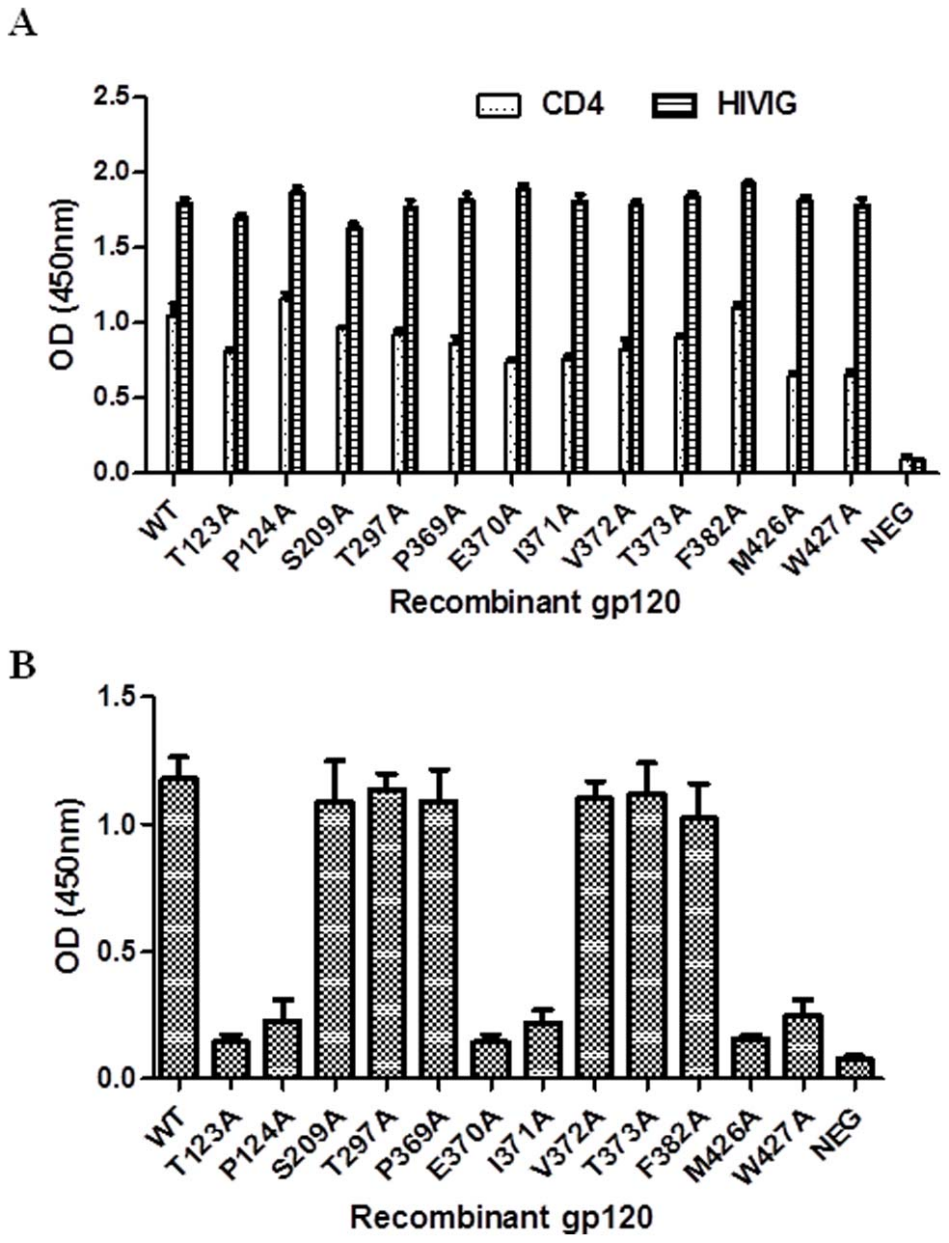
Mapping of m36-binding residues by point-mutagenesis. **A.** Reactivity of WT and mutant gp120 proteins with anti-HIV polyclonal antibody (HIVIG) and soluble CD4. **B.** Relative binding of m36 with WT and mutant gp120 proteins in ELISA. Wells were incubated with 0.1μg m36 in presence of soluble CD4.

Nine residues in three conserved domains of gp120 were identified by both the mimotope-based prediction and molecular docking, two (Thr123, Pro124) at the V1/V2 stem of C1 domain (designated site C1), five (Pro369, Glu370, Ile371,Val372, Thr373) in the C3 domain (site C3), and another two (Met426, Trp427) in the C4 domain (site C4). Although these three discontinuous sites are widely distributed in the linear sequence, they are in close proximity in the 3D structure of gp120 (Fig. 4B,4C). Therefore, we analyzed the potential importance of these residues by site-directed mutagenesis (alanine scanning). Three other residues (Ser209, Thr297, Phe382) mapped by the Mapitope alone were also included. As shown in Fig. 5A, all the recombinant gp120s carrying the specific point mutations bound to soluble CD4 (sCD4) and polyclonal anti-HIV antibodies (HIVIG) with strengths comparable to those of the wild-type gp120, indicating that they were correctly folded. Next, we tested the gp120 proteins for binding to m36 in the absence or presence of sCD4. M36 did not or only weakly react with the wild-type and the mutated gp120s without CD4 (data not shown). In the presence of sCD4, m36 bound to the wild-type gp120 and the gp120s with S209A, T297A, P369A, V372A, T373A or F382A substitution comparably (Fig. 5B). In contrast, mutation of the other six residues, including all four residues (T123, P124, M426, and W427) from the sites C1 and C4 and two residues (Glu370, Ile371) from the C3 site, largely reduced the reactivity of m36, suggesting that these residues play a critical role in gp120 interaction with m36 in the presence of CD4.

### M36-gp120 interaction

**Figure 6:**
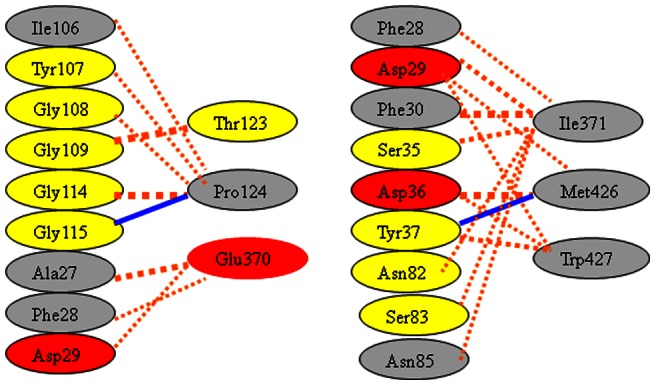
A schematic representation of the m36-gp120 interaction analyzed by the program “Protein Interface Analysis”. The orange dotted lines denote non-bonded interactions and blue lines denote hydrogen bonds.

The interactions between m36 and the defined six gp120 residues were further determined by the program “Protein Interface Analysis”. The docking model showed that the CDRs of m36 interact with the gp120 forming two hydrogen bonds and a number of non-bonded contacts (Fig. 6). In gp120, the m36-binding residues are located mainly in two domains, the bridging sheet and the CD4-binding loop. In m36, the non-bonded interactions are mostly caused by the residues from CDR1 (Ala27, Phe28, Asp29, Phe30, Ser35, Asp36 and Tyr37) and CDR3 (Ile106, Tyr107, Gly108, Gly114 and Gly115). Tyr37 in the CDR1 and Gly115 in the CDR3 of m36 create two hydrogen bonds against the carboxyl oxygen atoms of Met426 (distance, 2.39 Å) and Pro124 (distance, 1.99 Å), respectively.

## Discussion

This work was aimed at mapping the fine epitope of m36, a highly potent human antibody domain-based inhibitor of HIV-1 entry targeting the gp120 of HIV-1 Env. To achieve this goal we integrated three powerful approaches including mimotope-based computational prediction, molecular docking and site-directed mutagenesis. Three conserved gp120 regions comprised of six key residues (site C1: Thr123 and Pro124; site C3: E370A and I371A, and site C4: M426A and W427A) were identified that might interact with m36. They overlap with or are in close proximity to the CD4- and coreceptor-binding sites of gp120. Our results provide insights into the mechanisms of m36-mediated potent and broad inhibition of HIV-1 infection and will help in our future development of m36 as a novel class inhibitor of HIV-1 entry.

The cell entry of HIV-1 is mediated by its trimeric Env complex composed of surface subunit gp120 and transmembrane subunit gp41[23]–[26]. Binding of the cell receptor CD4 induces large conformational changes in gp120, resulting in the exposure and/or formation of the coreceptor-binding site that enables binding of gp120 to a coreceptor, either CCR5 or CXCR4. Subsequently, the fusion machinery of gp41, which is originally sheltered by gp120, is activated thus bringing the viral and cellular membranes into close proximity for fusion[27]–[28]. The entry steps offer attractive targets for antiviral agents, including gp120- or gp41-specific antibodies. Previous studies demonstrated that both CD4- and coreceptor-binding sites of gp120 are targeted by antibodies, some of which possess highly potent and broad inhibitory activity[10], [29]–[31]. Compared to antibodies that block CD4 binding (CD4bs antibodies), several antibodies targeting the CD4-induced coreceptor-binding sites (CD4i antibodies) are generally less effective in neutralizing HIV-1, such as 17b and X5[5], [32]. Interestingly, their neutralization is inversely correlated with antibody size [5]. For example, Fabs (with a size of 50–60 kDa) neutralize viruses generally better than their IgG formats (∼150 kDa for a, IgG1) and scFvs (20–30 kDa) could be more potent than their Fab formats. It was proposed that size-dependent neutralization could be due to steric restriction for antibody access to CD4i epitopes or the available space between the virus and the target cell surface after CD4 binding is not enough to accommodate a whole antibody molecule [5]. Therefore, it is conceivable that the smallest independently folded antibody domains (11–15 kDa) exhibit potent and broad neutralizing activity by targeting hidden conserved structures that are not accessible by larger antibodies. In agreement with this hypothesis, several single heavy chain variable domains (referred to as V_H_Hs) were successfully isolated from llama immunized with HIV-1 gp120 and characterized as potent HIV-1 entry inhibitors through interfering with virus binding to CD4 [15]. As the first reported engineered antibody domain (eAd) with a size of ∼15 kDa, m36 possesses extraordinarily potent activity in inhibiting genetically diverse HIV-1 isolates thus showing great potentials to overcome the disadvantages of those larger antibodies [18]. Based on the significantly increased reactivity with gp120 upon CD4 binding, we proposed that m36 might bind to a conserved CD4i structure [18]–[19]. Indeed, our studies here localized the binding residues of m36 in or near the co-receptor binding site. Apparently, it is constituted by three conserved motifs residing respectively in the V1/V2 stem (C1 site), C3 (C3 site) and C4 (C4 site) regions of gp120. The site-directed mutagenesis verified that a total of six residues, two from each of sites, are primarily involved in the formation of m36 epitope. In the 3D structure of gp120, the C1and C4 residues are located in the bridging sheet region and the C2 residues are within the CD4-binding loop (β15-α3 excursion)[23]. Notably, all the characterized m36-binding residues in gp120 are highly conserved among all HIV-1 isolates, explaining the broad-spectrum HIV-neutralizing activity by m36.

The molecular architecture of HIV-1 Env “spike” and the associated conformational changes upon the binding of CD4 or antibodies have been finely analyzed by using cryo-electron tomography[33]-[38], which reveal three distinct quaternary conformations of trimeric Env. A “closed” conformation is found when trimeric Env is unliganded or bound to the CD4bs antibodies VRC01,VRC02, or VEC03, characterized by the close positioning of adjacent V1V2 loops at the apex of the spike[34]–[35]; A “partially open” conformation is observed when Env is bound by the CDbs antibody b12, defined by a slight outward and rotational displacement of the gp120 monomers with respect to the central axis of the spike[34]; Third, an “open” Env structure is observed upon binding of soluble CD4 or the CD4i antibody 17b, featured by a quaternary conformation with large rearrangements of both gp120 and gp41[33]–[35]. Very recently, we determined the structures of m36 or m36/sCD4 complexed to the trimeric Env of native HIV-1_Bal_ virions by cryo-electron tomography, and showed that binding of the Env to m36 results in an “open” quaternary conformation similar to that seen with binding of soluble CD4 or 17b[19]. The structure of m36-bound Env displays an area of extra density at the apex of the spike, near the coreceptor binding site, and the footprint of m36 binding on gp120 is near the base of the V3 loop in a location similar to that of antibodies 17b and X5. Due to the low resolution of cryo-electron-based structures, it is impossible to discern the binding residues for a specific antibody. Interestingly, our studies discovered that m36 targets the face of the bridging sheet critical for CD4 binding (Fig. 4), whereas the epitopes for 17b and X5 reside on the opposite face of the bridging sheet[23], [39]. However, it was not surprised by the identification of the receptor CD4-binding residues, including those in the CD4-binding loop as the partial m36 epitope in gp120. Indeed, our previous studies showed that m36 could react with several gp120 proteins in absence of CD4 molecule, which are derived from different HIV-1 isolates[18]–[19]. Interestingly, our studies also demonstrated that a single Gln(Q) to Glu(E) mutation in the FR2 region of m36 could abolish the CD4-dependence for its reactivity to gp120[40]. We have verified that m36 does strongly bind to some gp120 proteins not depending on the addition of CD4 (data not shown). Therefore, both the receptor- and coreceptor-binding residues of gp120 may be involved in the formation of m36 epitope.

Taken together, these results provide a structural basis underlying the binding and neutralizing activity of m36. A crystal structure of m36 in complex with gp120 in the absence and/or presence of soluble CD4 would be helpful to further characterize the m36 epitope but unfortunately we have not been successful so far to grow good quality crystals of m36-gp120-CD4 complexes.

## Materials and Methods

### Expression and purification of m36

The Flag-tagged human domain antibody m36 was expressed in *E. coli* HB2151 as described previously [18]. In brief, the bacterial pellet was collected after centrifugation at 5,000 *g* for 10min and resuspended in PBS (pH 7.4) containing 0.5 million-unit polymixin B (Sigma-Aldrich). After 30 min incubation with rotation at 50 rpm at room temperature, it was centrifuged at 25,000 *g* for 25 min at 4°C. The supernatant was used for purification of m36 by immobilized metal ion affinity chromatography by using Ni-NTA resin (Qiagen, Valencia, CA) according to manufacturer’s protocols.

### Peptide library and biopanning

A random phage display library (Ph.D-12), wherein the displayed peptides (12-mer) are fused to the N terminus of gIII protein, was purchased from New England Biolabs (Beverly, MA). The complexity of the library is estimated to be 2×10^9^ which is sufficient to encode most peptide sequences. Affinity selection of the phage clones from the library was performed according to the manufacturer’s instructions. Briefly, 96-well plates (Costar, NY, USA) were coated with 100 µL of 0.1 µg/mL m36 in 0.1 M NaHCO_3_ buffer (pH 8.6) at 4°C overnight. The coated wells were blocked with 0.5% BSA in NaHCO_3_ buffer for 1 h at 37°C and then washed 6 times with TBS-T (TBS+0.1% Tween-20). The phage library (1×10^11^) was added into the coated wells and incubated at room temperature for 1 h. The wells were extensively washed with TBS-T for 10 times and the bound phages were eluted with 0.2 M Glycine-HCl (pH 2.2) containing 1 mg/ml BSA and neutralized with 1 M Tris-HCl (pH 9.1). The eluted phages were amplified by infecting log-phage *E. coli* ER2738, and then concentrated by polyethylene glycol (PEG) precipitation. The phages were tittered and submitted to the second round of selection. After 3–4 rounds of selection, the selected phage clones were identified by single-strand DNA sequencing and the encoded peptide sequence (mimotope) were deduced.

### Phage ELISA

To verification the positive phage clones, an indirect phage ELISA was performed. Briefly, a 96-well microtiter plate was coated with m36 at 100 ng/well and blocked by 0.5% BSA. Affinity-selected phages were first amplified in 2 mL ER2738 culture and then added 100 ul diluted culture supernatant to the coated wells. The plate was incubated for 1 h at room temperature and then washed three times with TBS-T (0.5% Tween 20). HRP-conjugated murine anti-M13 antibodies (Sigma) were added to the wells and incubated at 37°Cfor 1 h. The wells were extensively washed with TBS-T and reacted with TMB substrate (Sigma). Absorbance at 450 nm was measured with an ELISA reader (BioRad).

### Prediction of m36 binding residues by Mapitope

The potential binding residues of m36A were computationally predicted by the Mapitope algorithm[41]–[42], which is validated for efficient mapping of the conformation-dependent binding sites or epitopes based on the affinity-selected mimotopes by a number of studies[20]–[22]. In principle, the algorithm of Mapitope is based on the assumption that the epitope is separated into several amino acid pairs (AAP) contributing for the binding to the antibodies and the entire set of peptides is enriched with AAP which mimic the genuine epitope. The pairs of residues significantly overrepresented in the panel of peptides were identified in comparison to their expected frequencies. Mapitope then searches for patches on the surface that are enriched with these pairs. The resulting patches are presented on the 3D protein structure. The number of standard deviations above randomness for a given pair is defined as the statistical threshold (ST). Once the most frequent AAPs are identified, the algorithm seeks the pairs for a selected distance (D) value on the surface of the antigen and attempts to link them into clusters.

### Homology modeling and molecular docking of m36

Discovery Studio 3.5 (DS 3.5, Accelrys Software Inc., San Diego, CA) Modeler block was used to construct a homology model of m36. Heavy chain coordinates from antibodies with PDB codes 3E0T, 2NZ9, and 2GCY were used as templates for structure alignments to produce m36 homology models. All the 3 CDR loops in the m36 VH domain model were identified against antibody database, and reconstructed using the Model Antibody Loops protocol and subjected to Loops refinement protocol in DS 3.5. The final VH model was validated by using Ramachandran plot and Verify protein (Profiles-3D) protocol.

For protein-ligand docking, 3D structure of gp120 (PDB code: 2NXY) was retrieved from the PDB[43] and initialized as receptor molecules with the DS 3.5 tool “Protein Preparation”. All the possible torsion angles in ligand molecules (m36) were set to rotate freely. The flexible molecular docking of m36 VH onto gp120 model was accomplished using ZDOCK protocol in DS 3.5 with high predictive accuracy Generic Algorithm (GA) parameters. For each gp120 ligand, ten ligand poses were generated from docking experiment and evaluated with ZDOCK score function taking into account factors such as H-bonding energy, van der Waals energy and ligand torsion strain. The ligand poses with ZDOCK score above 11 were refined and analyzed using Rdock in DS 3.5. The top ranked ligand poses for gp120 were used for further study. The molecular interactions between m36 and gp120 were analyzed using the program “Protein Interface Analysis”.

### Site-directed mutagenesis

A vector (pCMV-gp120) expressing HIV-1_NL4-3_ gp120 was first constructed by PCR cloning. Briefly, the prime start DNA polymerase (Takara, Dalian, China) was used to extend a pair of mutated primers that introduce a stop codon (UAG) at the end of gp120 sequence by using the plasmid (pCMV-Env NL4-3 gp160) as a template. The PCR product was then digested with Dpn-I (NEB, Beverly, MA) to remove the methylated parental DNA. DNAs containing the desired mutations were then transformed into E. coli DH5α, followed by plating on selective LB plates (Ampicillin 100 µg/mL). A panel of site mutations corresponding to the predicted gp120 residues were generated based on the vector pCMV-gp120 as the template similarly. All the mutation were verified by DNA sequencing, and the protein expression was confirmed by Western-blot.

### Reactivity of m36 with HIV-1 gp120

To analyze the binding activity of m36 with the wild-type or mutated HIV-1_NL4-3_ gp120, 293T cells were transfected by a specific plasmid. The cells were then cultivated for 12 h in Optim-DMEM medium. The culture supernatant containing the expressed gp120 proteins were collected for ELISA assay. In brief, the 96-well microtiter wells were coated with 100 µL of culture supernatant overnight and blocked for 2 h with 5% BSA in PBS, followed by five washes with PBS-T. At first, the reactivity of soluble CD4 (2 µg/mL) or HIVIG (1 µg/mL) was tested to assess the binding capacity of the wild-type and mutated gp120. The reactivity of m36 (1 µg/mL) was then tested in the presence or absence of sCD4. All the reactions were placed at 4°C overnight. After three washes, the wells were incubated with 1 µg/mL either mouse anti-His tag mAb (for CD4; MBL, Woburn, MA), or mouse anti-Flag tag mAb (for m36; MBL, Woburn, MA), or goat anti-human IgG polyclonal antibodies (for HIVIG). The wells were washed 5 times with PBS-T and the TMB substrate (Sigma, St. Louis, MO, USA) was added. The absorbance was measured at 450 nm.
